# Value of apparent diffusion coefficient on MRI for prediction of histopathological type in anal fistula cancer

**DOI:** 10.1097/MD.0000000000033281

**Published:** 2022-04-07

**Authors:** Shinji Yamamoto, Keiji Yonezawa, Naoki Fukata, Koji Takeshita, Makoto Kodama, Tetsuro Yamana, Shigeru Kiryu, Yukinori Okada

**Affiliations:** a Department of Radiological Technology, Tokyo Yamate Medical Center, Tokyo, Japan; b Department of Medical Science, Suzuka University of Medical Science, Graduate School of Medical Science, Mie, Japan; c Department of Radiology, Tokyo Yamate Medical Center, Tokyo, Japan; d Department of Pathology, Tokyo Yamate Medical Center, Tokyo, Japan; e Department of Colorectal Proctology, Tokyo Yamate Medical Center, Tokyo, Japan; f Department of Radiology, International University of Health and Welfare Narita Hospital, Chiba, Japan; g Department of Radiology, Tokyo Medical University, Tokyo, Japan.

**Keywords:** anal fistula cancer, apparent diffusion coefficient, histopathological type

## Abstract

The main histopathological types of anal fistula cancers are mucinous adenocarcinoma and tubular adenocarcinoma. The purpose of this study was to investigate the utility of the apparent diffusion coefficient (ADC) value in magnetic resonance imaging (MRI) to determine the histopathological type of an anal fistula cancer, and to investigate the relationship between ADC values and histopathological type (mucinous type or tubular carcinoma), clinical information, and surgical findings. We retrospectively identified 69 patients diagnosed with anal fistula cancer at our hospital from January 2013 to December 2021. Among them, we selected the patients diagnosed using the same 1.5-T MRI machine, underwent surgery, and a pathological sample was obtained during the operation. Finally, these 25 patients were selected for the analysis since they underwent the imaging scan using the same MRI machine. The ADC value was compared between mucinous and tubular adenocarcinomas, and between tumors at the Tis-T1-T2 and T3-T4 stages. Finally, 25 patients were selected. The mean age of the 25 patients included in the analysis was 60.8 ± 13.3 years and all were males. The median ADC of anal fistula cancers was 1.97 × 10^–3^ mm^2^/s for mucinous adenocarcinomas and 1.36 × 10^–3^ mm^2^/s for tubular adenocarcinomas; this difference was statistically significant (*P* < .01). Furthermore, the median ADC was 1.62 × 10^–3^ mm^2^/s for tumors in Tis-T1-T2 stages and 2.01 × 10^–3^ mm^2^/s for T3-T4 tumors (*P* = .02). The ADC value in MR images may predict the histopathological type and depth of anal fistula cancers. Also, the different ADC values between Tis-T1-T2 and T3-T4 tumors could help predict the classification of progression.

## 1. Introduction

Cancer arising from a fistula in the rectal-anal region (anal fistula cancer) is a malignant type of tumor associated with long-term anal fistulas and is reported to account for approximately 2% to 3% of colorectal cancers.^[1,2]^ The World Health Organization states that anal fistula cancers originate from existing anal glands and fistula tracts.^[3]^ The early diagnosis of anal fistula carcinoma is difficult because of the lack of early clinical symptoms due to its occurrence in a complex and intricate anal fistula tract.^[4]^

The histopathological types of anal fistula cancer are classified as mucinous adenocarcinoma, tubular adenocarcinoma, adenosquamous carcinoma, and squamous cell carcinoma. In a review of the clinical pathology and treatment results of 42 cases of anal fistula cancer at our hospital, the tumors identified were 32 mucinous adenocarcinomas, 8 tubular type adenocarcinomas, 1 adenosquamous carcinoma, and 1 squamous carcinoma.^[2]^ In a review of the histopathology of 75 cases of anal fistula cancer in Crohn disease (CD), 51 were mucinous adenocarcinomas and 17 tubular type adenocarcinomas.^[5]^

Abdominoperineal resection is the first line of treatment for anal fistula cancers.^[6]^ However, this surgery results in permanent colostomy, which significantly reduces the quality of life. In addition, extensive lymph node dissection may cause lymph node edema as a complication, and it is difficult to predict small lymph node metastases from preoperative imaging. Pre- and postoperative chemoradiotherapy are effective in rectal cancer, and the indications for anorectal-sparing surgery are expanding^[7,8]^; however, few reports focused on chemoradiotherapy specifically for anal fistula cancer, and its therapeutic efficacy for these tumors remains unknown. Tubular adenocarcinomas are expected to respond to chemoradiotherapy, whereas the mucinous ones are resistant to these therapies.^[6,9,10]^ Therefore, determining the histopathology of an anal fistula cancer before treatment is critical.

Magnetic resonance imaging (MRI) findings in anal fistula cancers reportedly show a mesh-like contrast pattern with a full, contrasted tumor on spin-echo MRI, fluid retention without a fibrotic capsule, and contrasted tumor margins.^[11]^ T1- and T2-weighted images show heterogeneous high signal intensity.^[11]^ Such imaging findings may reflect the characteristic features of mucinous carcinoma as a cystic tumor with a protein component within multifocal septal walls.^[12]^ However, no previous report examined in detail the differences in MRI findings between mucinous and tubular adenocarcinomas.

The purpose of this study was to investigate the utility of the ADC value in MRI for the identification of the histopathological type of these tumors, and examine the relationship between ADC values and histopathological type (mucinous type or tubular carcinoma), clinical information, and surgical findings.

## 2. Methods

This study was based on a retrospective, cohort design; it was conducted in a single-center (Tokyo Yamate Medical Center) and approved by the Ethics Committee of our hospital (Application No. J-103, at April 8, 2021, approved change application form at March 25, 2023 Application No. J-171). Due to its retrospective nature, it was not possible to obtain prior informed consent from the patients; we used an opt-out form that was posted on the hospital website and at the reception desk of the Department of Radiology.

### 2.1. Case selection

Between January 2013 and December 2021, 69 cases of anal fistula cancer occurred in our hospital. These patients underwent surgery, and surgical pathological samples were obtained. Patients with concurrent colorectal cancer were excluded. Among them, we selected the patients diagnosed with anal fistula cancer using the same 1.5-T MRI machine, and these subjects were included in the analysis.

### 2.2. MRI protocol

The MRI scans were performed with a 1.5-T MRI system (MAGNETOM Aera, Siemens Healthcare, Erlangen, Germany), using an anal fistula protocol (Table 1). A noncontrast-enhanced MRI was used to obtain the images. All patients underwent diffusion-weighted imaging (DWI), apparent diffusion coefficient (ADC) testing, T1-weighted (T1WI), and T2-weighted (T2WI) MRI.

**Table 1:** Sequence parameters for magnetic imaging of anal fistulae.

Protocol	T2WI tse	DWI	T2WI space 3D	FS T2WI tse f-Dixon	T2WI tse	T1WI fl3d Dixon pre (DCE)
Orientation	Sag	Tra	Tra	Obl-tra	Obl-cor	Obl-tra
TR (ms)	3400	5000	1100	3080	4179	7.13
TE (ms)	89	63	95	87	87	2.39/4.77
Slice thickness (mm)	3.5	6	–	3	3	3
FOV read (mm)	250	350	250	250	250	250
Slices	20	30	–	30	30	–
Distance factor (%)	20	10	–	20	20	–
Averages	1	–	1.5	1	2	1
Fat- suppressed	–	SPAIR	–	Fast Dixon	–	Dixon
*b*-value		0, 50, 800				
Scan time (s)	95	145	223	136	154	24

3D = 3-dimensional, Cor = coronal, DCE = dynamic contract enhancement, DWI = diffusion-weighted imaging, fl3d = flash 3-dimensional, FOV = field of view, FS T2WI = fat-suppressed T2-weighted magnetic resonance imaging, Obl = oblique, Sag = sagittal, SPAIR = spectral attenuated inversion recovery, T1WI = T1-weighted magnetic resonance imaging, T2WI = T2-weighted magnetic resonance imaging, TE = echo time, TR = repetition time, Tra = transverse, tse = turbo spin echo.

### 2.3. Image evaluation

The MR images were evaluated by 2 radiologists with more than 20 years of experience (KT and SK); one is also specialized in anal fistula MRI diagnosis (SK). Both radiologists received information about the patient prior diagnosis of anal fistula cancer and the location of the lesion on MRI, and agreed on the area of the lesion.

**Figure 1.**
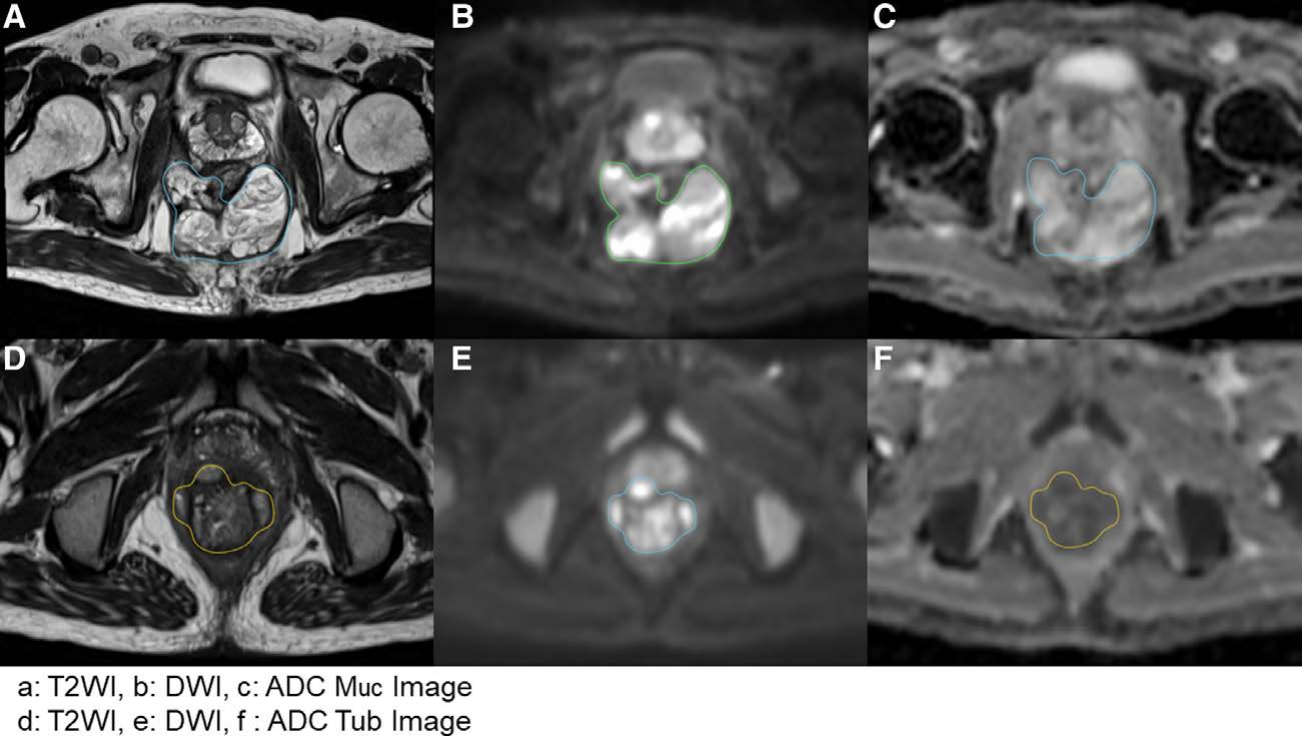
MRI images. (a–c) T2WI, DWI, and ADC images of a mucinous adenocarcinoma; (d–f) T2WI, and DWI, and ADC images of a tubular adenocarcinoma. ADC = apparent diffusion coefficient, DWI = diffusion-weighted imaging, MRI = magnetic resonance imaging, T2WI = T2-weighted imaging.

The Digital Imaging and Communications in Medicine (DICOM) images were imported into the open-source image processing tool OsiriX (OsiriX Foundation, Geneva, Switzerland). The 2 radiologists used the MR images acquired with the anal fistula protocol as a reference to select the region of interest with diffusion restriction on DWI as the lesion area (DWI-region of interest) on the corresponding ADC image (Fig. 1).

### 2.4. Clinical findings

The clinical information was obtained from electronic medical records. Age at diagnosis, sex, and presence or absence of CD were included in the data for the analysis.

### 2.5. Surgical and pathological findings

The surgical findings were obtained from the records of the primary surgeon. The evaluation variables were the surgical technique and the presence or absence of lymph node metastases. The pathological findings were obtained from the diagnostic records of the pathologists. The evaluation variables were histopathological type, histological differentiation, depth, lymphatic invasion, venous invasion, and staging. Since the histopathological types of anal fistula cancer have a spectrum, the components of mucinous and tubular adenocarcinoma were compared. In accordance with the World Health Organization Colorectal Cancer Code, tumors with a prevalence of mucinous findings were classified as mucinous adenocarcinomas, and those with mainly tubular cells as tubular adenocarcinomas.

### 2.6. Statistical analysis

Statistical analysis was conducted with Easy-R (EZR), developed at the Omiya Medical Center of Jichi Medical University Hospital.^[13]^

The Mann–Whitney *U* test was used to compare the radiological characteristics (median ADC) between different clinical, surgical, and pathological groups. A receiver operating characteristic (ROC) curve analysis was used to calculate the cutoff values and area under the curve showing the test efficacy. The Fisher test was used to examine the distributional bias. For all statistical tests, *P* < .05 was set as the level of statistical significance.

## 3. Results

### 3.1. Patients

Finally, 25 patients were selected. The mean age of the 25 patients included in the analysis was 60.8 ± 13.3 years and all were males. All patients underwent rectal amputation; 24 of them also received pelvic lymphadenectomy. The histological types were 17 mucinous adenocarcinomas, 7 tubular adenocarcinoma, and 1 unclassifiable. Six patients had CD. Lymph node metastases were observed in 3 patients; as for the staging, Tis-T1-T2 and T3-T4 tumors were found in 10 and 15 patients, respectively. The median ADC was 1.80 (range: 1.28–2.27) × 10^–3^ mm^2^/s.

### 3.2. Comparison between surgical and pathological findings

#### 1.3.2. Histopathology.

The median ADC was 1.97 × 10^–3^ mm^2^/s in mucinous adenocarcinomas and 1.36 × 10^–3^ mm^2^/s in tubular adenocarcinomas, and there was a significant difference between these 2 groups (*P* = .005). In the ROC analysis, the area under the curve was 0.857, with a 95% confidence interval of 0.627 to 1, when the ADC cutoff value was set at 1.74. There were no statistically significant differences in age and lymph node metastases between patients with and without CD.

#### 2.3.2. Depth.

**Figure 2.**
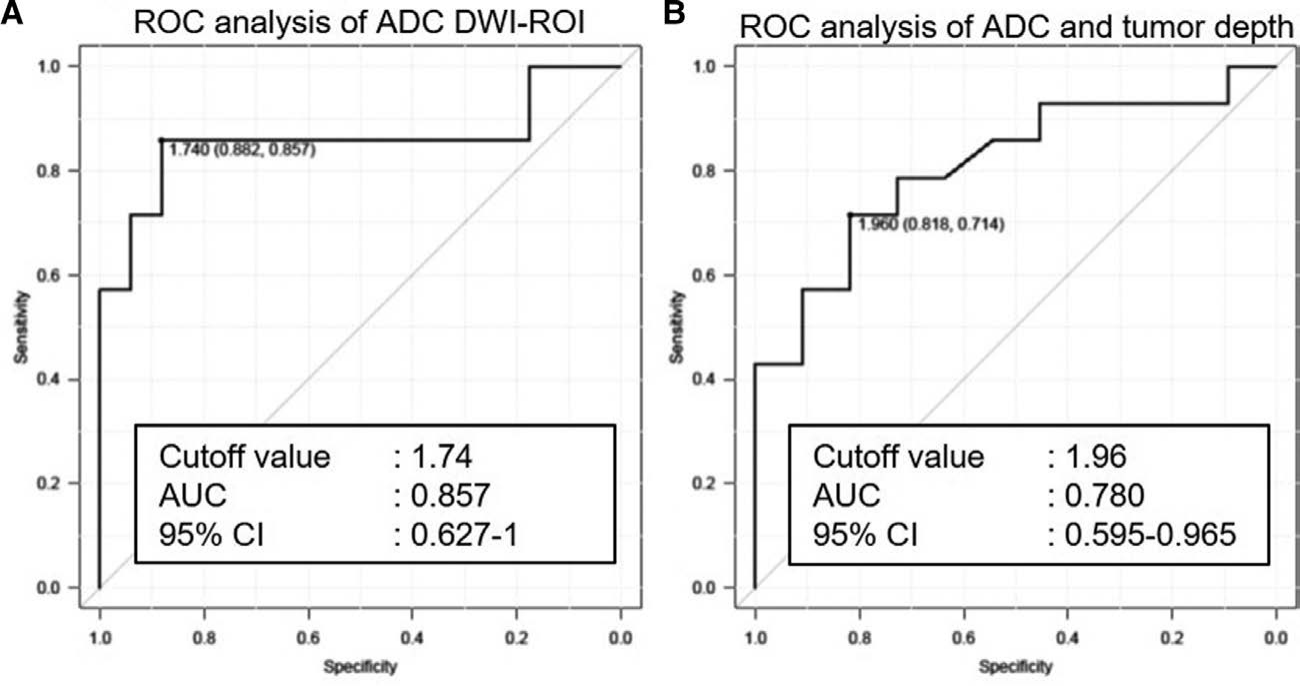
ROC analysis of ADC values to determine the tumor histopathological type (left) and depth (right). ADC = apparent diffusion coefficient, AUC = area under the curve, CI = confidence interval, DWI-ROI = diffusion-weighted imaging-region of interest, ROC = receiver operating characteristic.

The median ADC was 1.62 × 10^–3^ mm^2^/s for the Tis-T1-T2 group and 2.01 × 10^–3^ mm^2^/s for the T3-T4 group, and the difference between the 2 groups was statistically significant (*P* = .02). In the ROC analysis, the area under the curve was 0.780, with a 95% confidence interval of 0.595 to 0.965, when the cutoff value of ADC was set at 1.96 × 10^–3^ mm^2^/s. There were no significant differences in age, presence or absence of CD, or lymph node metastases between these 2 groups. Table 2 and Figure 2 summarize the above results.

**Table 2:** Comparison between mucinous adenocarcinoma and tubular adenocarcinoma.

Histological	Muc: n = 17	Tub: n = 7	*P* value
Age at cancer diagnosis	64	62	.070
ADC mean (×10^–3^ mm^2^/s)	1.88	1.39	.005
CD	3	3	
Tumor depth	Tis-T1-T2	T3-T4	*P* value
Age at cancer diagnosis	58	67	.760
ADC mean (×10^–3^ mm^2^/s)	1.62	2.01	.020

ADC = apparent diffusion coefficient, CD = Crohn disease, Muc = mucinous adenocarcinoma, Tub = tubular adenocarcinoma.

## 4. Discussion

This study compared the imaging findings in anal fistula cancer in different surgical and pathological groups. The histopathological classification was 68% (17/25) mucinous and 28% (7/25) tubular adenocarcinomas. In a study of 42 cases of anal fistula cancers at our institution, we found 76% (32/42) mucinous and 19% (8/42) tubular adenocarcinomas.^[2]^ In a study of 75 cases including anal fistula cancers related to CD, the histopathological types were reported as 68% (51/75) mucinous and 23% (17/75) tubular adenocarcinomas.^[9]^ The findings of the present study were similar to the results of these reports, with frequencies of mucinous and tubular adenocarcinomas in anal fistula cancers of approximately 70% and 20%, respectively.

There are few studies on the treatment of anal fistula cancer, and reports are predominantly case reports. One case report described the operation and adjuvant FOLFOX6 chemotherapy in a patient with mucinous adenocarcinoma (anal fistula cancer).^[14]^ In 5 cases of anal fistula cancer (4 mucinous adenocarcinomas and 1 nonmucinous adenocarcinoma), 1 patient (T3N1M0) received neoadjuvant chemoradiotherapy, 1 patient (T4N0M0) received chemotherapy, 1 patient underwent an operation for mucinous adenocarcinoma,^[15]^ 2 patients (with mucinous adenocarcinoma) received radiation and chemotherapy, and 1 patient with lymph node metastasis died following distant metastasis.^[16]^ From these reports, it is clear that the treatment strategy for anal fistula cancer is not established overseas. In Japan, there are few reports on anal fistula cancer. The standard treatment for anal fistula cancer is surgery. Operation and abdominoperineal resection was reportedly mainly performed in 15 patients with anal fistula cancer.^[6]^ Furthermore, the protocol for chemotherapy is not established. There are some reports on the effects of chemotherapy and pathological type of colon cancer. It has been reported that mucinous carcinoma has a poor response to chemotherapy.^[6]^ It was reported that mucinous, colon, and rectal carcinoma showed poor response to 5-fluorouracil in 135 patients.^[6,9]^ In a study comparing 49 patients with mucinous colorectal carcinoma to 206 patients with non-mucinous colorectal carcinoma, those with mucinous colorectal carcinoma showed poor response to irinotecan and/or oxaliplatin and had poor prognoses.^[10]^ This study suggested that treatment response may be poor in patients with mucous carcinoma. In 1 review article, the utility of chemoradiotherapy was unclear.^[17]^ However, histological type determination may become important in selecting the treatment strategy for anal fistula cancer, especially chemotherapy.

MRI is typically used in the imaging diagnosis of anal fistula cancer; in particular, DWI shows the tumor with high signal intensity. Furthermore, the ADC value is a parameter that can be used in clinical practice as a quantitative imaging biomarker.^[18]^ In the present study, the difference in ADC values between mucinous and tubular adenocarcinomas reflects the pathological differences between these 2 types of tumor. Therefore, preoperative MRI is necessary, and the ADC value may suggest the histopathological diagnosis. In this study, non-contrast-enhanced MRI was evaluated. Based on the pathological findings at our hospital, the histological difference between mucinous and tubular adenocarcinomas is that the former type tends to be edematous and with more stroma than the latter because of more mucus leaking into the stroma. In addition, mucinous carcinomas tend to have a sparse cell density due to the large amount of interstitium and may have fewer blood vessels than tubular adenocarcinomas. In the future, contrast-enhanced MRI may provide useful additional information for the histopathological diagnosis of anal fistula cancers.

This study has several limitations. First, the number of cases of anal fistula cancer was small because it is a rare disease arising from a fistula tract. Second, the study was retrospective, thus patient selection bias cannot be excluded. Third, it is difficult to completely define the area of anal fistula cancer compared with pathological findings on preoperative MRI images. Further studies are needed to increase the number of cases and examine the results in a multicenter setting.

## 5. Conclusion

The ADC values in preoperative anal MR images could distinguish the histopathological types of mucinous and tubular adenocarcinoma in an anal fistula cancer. Also, the different ADC values between Tis-T1-T2 and T3-T4 groups could help predict the classification of progression. Therefore, these results may provide useful information to decide the treatment strategy for these patients.

## Author contributions

**Conceptualization:** Shinji Yamamoto, Tetsuro Yamana.

**Formal analysis:** Koji Takeshita, Shigeru Kiryu.

**Investigation:** Keiji Yonezawa, Naoki Fukata, Koji Takeshita, Makoto Kodama, Shigeru Kiryu.

**Project administration:** Yukinori Okada.

**Supervision:** Yukinori Okada.

**Writing – original draft:** Shinji Yamamoto.

**Writing – review & editing:** Shinji Yamamoto, Tetsuro Yamana.

## References

[1] Santos MD Nogueira C Lopes C. Mucinous adenocarcinoma arising in chronic perianal fistula: good results with neoadjuvant chemoradiotherapy followed by surgery. Case Rep Surg. 2014;2014:386150. 25506029 10.1155/2014/386150 PMC4251890

[2] Sassa M Yamana T Ono T. Clinicopathological characteristics and clinical outcomes of anal fistula cancer in 42 patients. [Japanese]. Nippon Daicho Komonbyo Gakkai Zasshi. 2017;70:57–63.

[3] Welton ML Lambert R Bosman FT. Tumors of the anal canal. In: Bosman FT Carneiro F Hruban RH, ., eds. WHO Classification of Tumors of the Digestive System. Lyon: International Agency for Research on Cancer, 2010:185–193.

[4] Shwaartz C Munger JA Deliz JR. Fistula-associated anorectal cancer in the setting of Crohn’s disease. Dis Colon Rectum. 2016;59:1168–73. 27824702 10.1097/DCR.0000000000000700

[5] Kodama M Kobayashi D Iihara K. Adenocarcinoma within anorectal fistulae: different clinicopathological characteristics between Crohn’s disease-associated type and the usual type. Mod Pathol. 2019;32:314–25. 30206406 10.1038/s41379-018-0105-8

[6] Nakajima K Kobayashi A Koda T. Carcinoma associated with anal fistula: a clinicopathologics study in 15 patients. [Japanese]. Nippon Daicho Komonbyo Gakkai Zasshi. 2010;63:346–58.

[7] Kapiteijn E Marijnen CA Nagtegaal ID. Preoperative radiotherapy combined with total mesorectal excision for resectable rectal cancer. N Engl J Med. 2001;345:638–46. 11547717 10.1056/NEJMoa010580

[8] Sauer R Becker H Hohenberger W. Preoperative versus postoperative chemoradiotherapy for rectal cancer. N Engl J Med. 2004;351:1731–40. 15496622 10.1056/NEJMoa040694

[9] Negri FV Wotherspoon A Cunningham D. Mucinous histology predicts for reduced fluorouracil responsiveness and survival in advanced colorectal cancer. Ann Oncol. 2005;16:1305–10. 15857840 10.1093/annonc/mdi244

[10] Catalano V Loupakis F Graziano F. Mucinous histology predicts for poor response rate and overall survival of patients with colorectal cancer and treated with first-line oxaliplatin- and/or irinotecan-based chemotherapy. Br J Cancer. 2009;100:881–7. 19259089 10.1038/sj.bjc.6604955 PMC2661784

[11] Hama Y Makita K Yamana T. Mucinous adenocarcinoma arising from fistula in ano: MRI findings. AJR Am J Roentgenol. 2006;187:517–21. 16861558 10.2214/AJR.05.0011

[12] Zhu X Zhu TS Ye DD. Magnetic resonance imaging findings of carcinoma arising from anal fistula: a retrospective study in a single institution. World J Clin Cases. 2020;8:5159–71. 33269252 10.12998/wjcc.v8.i21.5159 PMC7674754

[13] Kanda Y. Investigation of the freely available easy-to-use software “EZR” for medical statistics. Bone Marrow Transplant. 2013;48:452–8. 23208313 10.1038/bmt.2012.244 PMC3590441

[14] Komornik FN Zafar M Karimi S. Anal adenocarcinoma arising from a fistula-in-ano: a case report. Cureus. 2022;14:e31339. 36514648 10.7759/cureus.31339 PMC9733786

[15] Leong FQ Chan DKH Tan KK. Anal adenocarcinoma can masquerade as chronic anal fistula in Asians. Ann Coloproctol. 2019;35:47–9. 30509019 10.3393/ac.2018.03.15 PMC6425245

[16] Yang BL Shao WJ Sun GD. Perianal mucinous adenocarcinoma arising from chronic anorectal fistulae: a review from single institution. Int J Colorectal Dos. 2009;24:1001–6. 10.1007/s00384-009-0657-7 19205706

[17] Tekbaş A Mothes H Settmacher U. Non-mucinous adenocarcinomas and squamous cell carcinomas of the anal region masquerading as abscess or fistula: a retrospective analysis and systematic review of literature. J Cancer Res Clin Oncol. 2022;148:1509–1522. 34338860 10.1007/s00432-021-03747-8 PMC9114013

[18] Horvat JV Bernard-Davila B Helbich TH. Diffusion-weighted imaging (DWI) with apparent diffusion coefficient (ADC) mapping as a quantitative imaging biomarker for prediction of immunohistochemical receptor status, proliferation rate, and molecular subtypes of breast cancer. J Magn Reson Imaging. 2019;50:836–46. 30811717 10.1002/jmri.26697 PMC6767396

